# Effect of Obesity on Knee and Ankle Biomechanics during Walking

**DOI:** 10.3390/s21217114

**Published:** 2021-10-27

**Authors:** Paolo Capodaglio, Michele Gobbi, Lucia Donno, Andrea Fumagalli, Camillo Buratto, Manuela Galli, Veronica Cimolin

**Affiliations:** 1 Orthopaedic Rehabilitation Unit and Clinical Lab for Gait Analysis and Posture, Ospedale San Giuseppe, Istituto Auxologico Italiano, IRCCS, Via Cadorna 90, 28824 Verbania, Italy; m.gobbi@auxologico.it (M.G.); a.fumagalli@auxologico.it (A.F.); 2 Department of Surgical Sciences, Physical and Rehabilitation Medicine, University of Torino, Via Giuseppe Verdi, 8, 10124 Torino, Italy; 3 Department of Electronics, Information and Bioengineering, Politecnico di Milano, Piazza Leonardo da Vinci 32, 20133 Milano, Italy; lucia.donno@polimi.it (L.D.); manuela.galli@polimi.it (M.G.); veronica.cimolin@polimi.it (V.C.); 4 Podartis srl, Via Erizzo 123/c, 31035 Crocetta del Montello, Italy; camillo.buratto@podartis.it

**Keywords:** obesity, gait analysis, knee and ankle biomechanics, walking, rehabilitation

## Abstract

The purpose of this retrospective study was to quantify the three-dimensional knee and ankle joint kinematics and kinetics during walking in young participants with different degrees of obesity and to identify the associated effects by stratifying the obese participants according to their BMI. Thirty-two young obese individuals (mean age 30.32 years) and 16 normal-weight age-matched individuals were tested using 3D gait analysis. Analysis of kinematic and kinetic data revealed significant differences in mechanics at knee and ankle joints in all the evaluated planes of movement. Compared to the healthy-weight participants, obese adults demonstrated less knee flexion, greater knee ab-adduction angle during the entire gait cycle and abnormalities at the knee flex-extension moment. At the ankle joint, reduced range of motion was observed together with a lower peak of ankle plantarflexor moment and power during terminal stance. These results provide insight into a potential pathway by which obesity predisposes a healthy adult for increased risk of osteoarthritis.

## 1. Introduction

Obesity elevates the risk for comorbidities, including musculoskeletal disorders such as osteoarthritis (OA), low back pain, soft tissue injury, tendinitis and plantar fasciitis [1,2,3]. Increased body mass, with increased forces across weight-bearing joints, has been causally implicated in many of these musculoskeletal conditions [4]. Forces on joint surfaces are increased during weightbearing activities, including walking. Increased body mass may augment risk of damage and injury to joint surfaces and other musculoskeletal structures with repetitive loading during weightbearing activities.

A deeper understanding about influence of obesity on gait biomechanics is being achieved [5,6,7,8]. However, the characterization of the kinematics and kinetics of walking using gait analysis in obese individuals is often inconsistent. While some studies reported that obesity induces slower velocity [3,6,9,10], reduced cadence [3,9,10], diminished stride length and single support time [2,3,6,10], lower swing time [10,11], increased stance time [6,10,11] and increased double support time [6,12], others failed in identifying differences as for velocity [2,11,12,13,14], cadence [11,12,15], step length [9,12,14], stride length [15], stance time [11], single and double leg support time and swing phase duration [9]. However, increased step width is reported in most papers [2,3,12,14,15]. Increased peak hip joint flexion [16], extension [10] and sagittal plane range of motion (ROM) [5] have been found. Conversely, no changes in hip joint sagittal plane ROM have been reported [16]. Finally, some papers describe an increased hip adduction during terminal stance and pre-swing and higher knee adduction in stance and swing [6,16] together with higher ankle plantarflexion and lower knee flexion [10] as compared to lean counterparts. It appears that investigations on the effects of obesity on walking using gait analysis have generated inconsistent results so far. Only few studies have reported frontal and transversal plane biomechanics of the knee joint at specific points during the gait cycle in obese subjects [17,18]. In addition, several differences among studies are present in terms of age, degree of obesity and methodological issues (i.e., the use of a treadmill mounted force plate to measure kinetic by Browning and Kram [15] or traditional gait analysis in level walking [2,3,4,5,6,7,8,9,10,11,12,13,14,16,17,18]).

Given the relative importance of mechanical factors and body weight in the development and progression of varus/valgus angular deformities of the knee and foot, as well as the long-term implications of increased risk of osteoarthritis, a deeper understanding of the effects of obesity on the kinematics and kinetics of gait is needed. Therefore, the main purpose of this study was firstly to quantify the three-dimensional knee and ankle joint kinematics and kinetics during walking using gait analysis in participants with obesity and secondly to identify the associated effects by stratifying the young obese participants according to their Body Mass Index.

## 2. Methods

### 2.1. Participants

For this retrospective study, the data came from all patients with obesity attending for an instrumented Gait Analysis during the years 2004–2018. If patients attended more than once during this period, then only data from their first visit were considered. Inclusion criteria were to be free from any acute musculoskeletal, neuromuscular, psychological and/or cardiopulmonary conditions able to significantly affect their walking abilities and postural control at the time of the experimental tests.

According to these criteria, a sample of 32 young obese individuals (OG, 15 male, 17 female, mean age 30.32 years, BMI > 30 kg/m^2^) admitted for a comprehensive multidisciplinary rehabilitation program at the Istituto Auxologico Italiano, Piancavallo (VB, Italy), were included in the study. Participants were classified into the three groups according to the BMI values (Class 1: BMI of 30 to <35 kg/m^2^; Class 2: BMI of 35 to <40 kg/m^2^; Class 3: BMI of 40 kg/m^2^ or higher).

A group of 16 normal-weight individuals (6 male, 10 female, mean age 31.07 years) recruited among the hospital staff served as control group (CG). Anthropometric and clinical features are reported in Table 1. All participants were required to sign a written informed consent form, in which the details of the experimental tests were reported. The study was carried out in compliance with the World Medical Association Declaration of Helsinki and its later amendments.

### 2.2. Data Collection and Processing

Gait and anthropometric data for each participant were collected in the Movement Analysis Lab of the San Giuseppe Hospital, Istituto Auxologico Italiano, Piancavallo (VB). Kinematic and kinetic parameters of gait were acquired by means of a 6-camera motion capture system (460 VICON, Oxford Metrics Ltd., Oxford, U.K.) with a sampling rate of 100 Hz and two force platforms (Kistler, CH). For a complete assessment, two TV camera video systems for videorecording were synchronized with the optoelectronic system and the platforms.

On each subject, a preliminary collection of the following anthropometric data was performed: body mass, height, anterior superior iliac spines (ASIS) distance (pelvis width), greater trochanter-ipsilateral ASIS distance (pelvic height), ASIS-ipsilateral medial malleolus distance (leg length), medial-lateral femoral condyles distance (knee diameter) and medial-lateral malleoli distance (ankle diameter). Thus, to assess the kinematics of each body segment, 22 spherical retro-reflective passive markers were carefully positioned on the participants’ skin at specific bony landmarks identified by manual palpation, according to the protocol proposed by Davis et al. [19]. Hence, subjects were required to walk barefoot along a 10 m walkway at their own natural cadence (self-selected speed). A trial was considered valid on the basis of good quality of the marker trajectories and included at least one cycle per limb with ground reaction forces.

A full gait cycle is defined as a periodic cycle involving two legs from the initial contact of one foot on the ground to the following occurrence of the same event with the same foot. Typically, a gait cycle is divided into two main phases. The stance phase, which represents approximately 60% of a gait cycle, starts when the foot strikes the ground and ends when it leaves the ground. The swing phase, which accounts for approximately 40% of the remaining gait cycle, starts when one foot leaves the ground and lasts until it touches the ground again. The initial contact marks the beginning of the stance phase and the toe-off marks the beginning of the swing phase. The data analysis for the gait analysis works on 3D trajectories of passive markers and relies on detecting the first heel impact event for each foot on the force platform.

**Figure 1:**
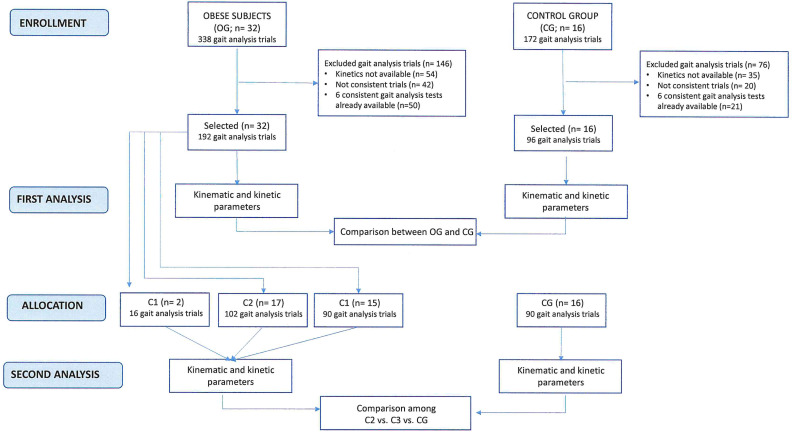
Flowchart of the experimental procedure.

Kinematic and kinetic data were collected for each participant from 6 valid gait trials in order to ensure the reproducibility of our results. The flowchart of the experimental procedure is displayed in Figure 1.

Collected data were thus exported by Vicon Polygon Application, Oxford, U.K., version 2.4, and processed using the dedicated software Vicon Polygon Application, Oxford, U.K., version 1.10.433.0, to assess specific kinematic and kinetic parameters with a focus on knee and ankle joints.

### 2.3. Kinematics

− Knee flexion angle (KFE-IC index), knee rotation angle on the transverse plane (KRot-IC index) and ankle dorsiflexion angle (ADP-IC index) at Initial Contact;
− Maximum knee ab-adduction in stance (MaxKAASt index) and in swing phase (MaxKAASw index);
− Maximum knee rotation angle on the transverse plane (MaxKRot index) in the whole gait cycle;
− Maximum ankle dorsiflexion (MaxADP index) and plantarflexion angles (minADP index) during the stance and swing phase, respectively;
− Dynamic range of motion (ROM) of the knee in the sagittal plane during stance (KFE-ROMSt index) and swing (KFE-ROMSw index) phases, separately calculated as the difference between the maximum and minimum flexion-extension angle reached on the sagittal plane in each phase;
− Dynamic range of motion for ankle dorsi-plantarflexion (ADP-ROM index) in the whole gait cycle.

### 2.4. Kinetics

− Maximum value of ankle plantarflexion moment in terminal stance (MaxADPMom index, N*m/Kg), first peak of knee abduction moment (MaxKAAMom index, N*m/Kg) and maximum value of knee extension moment (MaxKFEMom index, N*m/Kg) in the whole gait cycle;
− Minimum (minAP index, W/Kg) value in the first phase of stance and maximum (MaxAP index, W/Kg) ankle power during terminal stance.

All the kinetic parameters were reported both as normalized values respect to the subject’s body weight and as absolute values.

### 2.5. Statistical Analysis

Sample size was calculated based on a previous study [6] by using G. Power 3.1. We estimated that we needed 16 participants to detect a mean difference of 10.03° in knee max swing adduction, considering 90% power and two-sided alpha 0.05. As we had gait analysis tests of 32 obese individuals in our database, we decided to use data from all patients.

We considered all data separately acquired for left and right limb. First, the Kolmogorov–Smirnov test was used to verify the possible normal distribution of the estimated parameters. Since the normality hypothesis was satisfied, the mean value and standard deviation were considered for all the parameters. Then, the t-test for independent samples was used to verify the statistical difference between the parameters of the two groups (OG vs. CG). Then, to assess the presence of differences according to the class of obesity, univariate analysis of variance (ANOVA) was carried out followed by a post-hoc test among the different groups of obese participants and CG. In this analysis, as the C1 group was composed of just 2 participants, only C2 and C3 were included. For all the statistical tests, probabilities below 0.05 (*p* < 0.05) indicate rejection of the null hypothesis.

## 3. Results

Participant characteristics are presented in Table 1. There were no significant differences between OG and CG in terms of age and height; however, body mass (*p* < 0.05) and BMI (*p* < 0.05) were significantly higher in OG. In Table 2, the values of kinematic and kinetic parameters are displayed.

**Figure 2:**
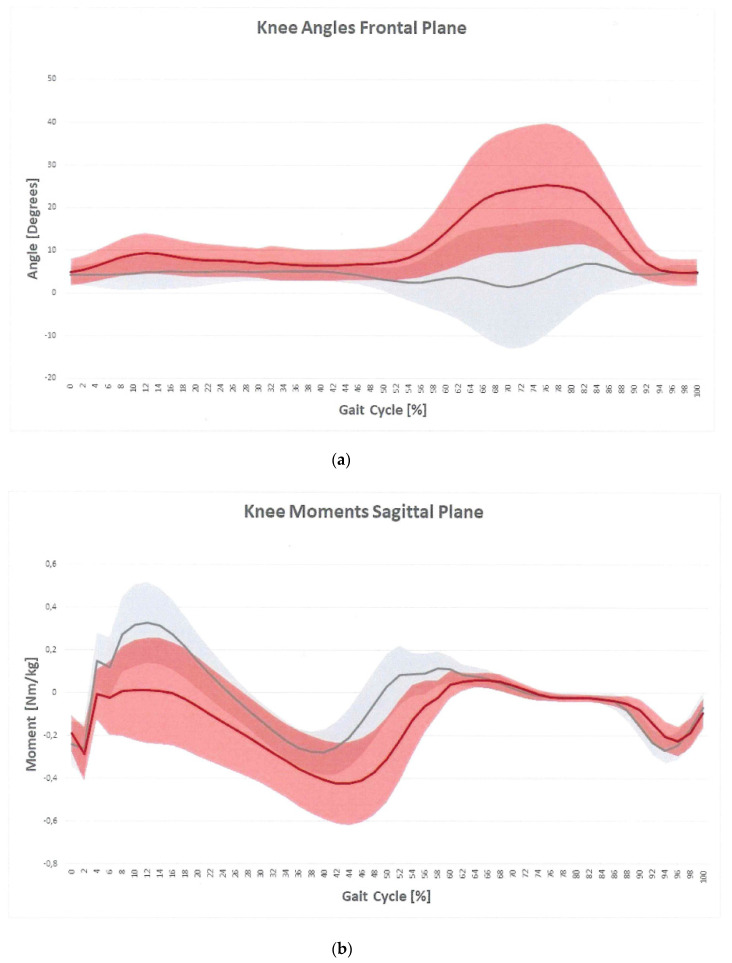
Knee angles plot on frontal plane (**a**) and knee moment plot on sagittal plane (**b**) for OG (red) and CG (gray). The vertical line is representative of toe-off.

**Figure 3:**
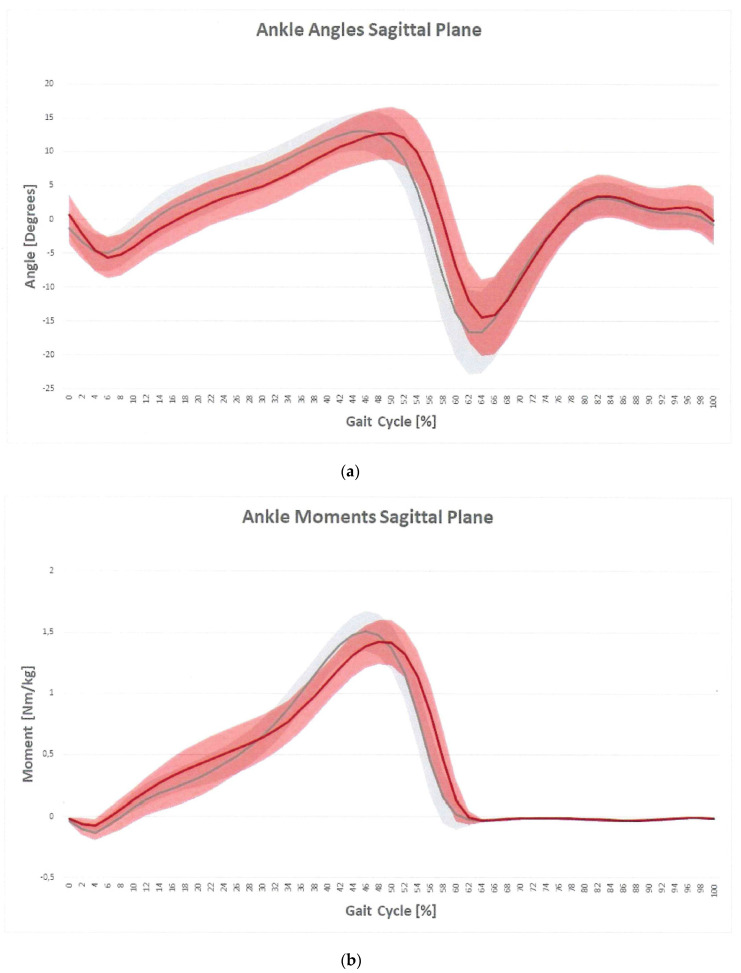
Ankle angles plot on sagittal plane (**a**) and ankle moment plot on sagittal plane (**b**) for OG (red) and CG (gray). The vertical line is representative of toe-off.

On the sagittal plane, OG maintained a relatively more extended knee throughout stance and swing, as confirmed by the FKE-ROMSt and KFE-ROMSw indices, lower in OG than CG. The knee flexion moment (MaxKFEMom index) was significantly lower in OG (Figure 2b). As for the ankle joint, kinematics showed reduced values of dynamic joint excursion (ADP-ROM index) in OG as compared to CG (Figure 3a). Participants who were obese had a significantly lower plantarflexion moment during late stance (MaxADPMom index) as compared to CG (Figure 3b). As for ankle power, both the minimum (minAP index) and the maximum value in terminal stance (MaxAP index) were significantly different compared to CG.

On the frontal plane, OG had a significantly higher adduction knee angle during the entire gait cycle (MaxKAASt and MaxKAASw indices) (Figure 2a). Frontal plane knee moment was not significantly different between groups.

On the transversal plane, OG revealed higher extra knee rotation at initial contact (KRot-IC index) compared to CG.

To assess the presence of differences according to the class of obesity, as the C1 group was composed of just two participants, only C2 and C3 were included. In the comparison between C2, C3 and CG, the following significant differences were found in some kinetic parameters:

- MaxKAAMom abs: C2: 51.3 ± 11.9 N*m (47.0; 55.6 N*m); C3: 72.5 ± 19.4 N*m (64.6; 80.3 N*m), CG: 33.2 ± 7.5 N*m (30.5; 35.9 N*m) (F2,87 = 2.59; *p* < 0.05).
- MaxADPMom abs: C2: 144.2 ± 29.7N*m (133.3; 155.1 N*m); C3: 172.7 ± 33.4 N*m (158.6; 186.8 N*m); CG: 89.7 ± 15.5 N*m (84.1; 95.3 N*m) (F2,86 = 69.87; *p* < 0.05).
- Max AP abs: C2: 309.2 ± 61.1 W (286.9; 331.6 W); C3: 367.5 ± 80.7 W (331.8; 403.2 W); CG: 202.7 ± 70.3 W (177.4; 228.1 W) (F2,84 = 38.93; *p* < 0.05).

Values in C3 were higher than C2, but no differences were observed when normalizing data compared to body weight. No other differences were found as for kinematics and kinetics.

## 4. Discussion

The purpose of this study was to identify differences in knee and ankle kinematics and kinetics during gait between young adults with normal weight and with different degrees of obesity.

Although the overall gait patterns of OG were qualitatively similar to those of the normal-weight group, several differences were observed. Specifically, during early stance, the obese subjects walked with a reduced knee flexion due to possible weakness of the knee extensors [18]. This result could be related to the lower knee extensor moment during early stance. Several hypotheses could explain why this gait abnormality was observed. Maintaining a straighter leg in early stance may be a compensation strategy for knee instability to decrease the moment arm acting on the knee in the sagittal plane. Alternatively, the extended knee angle could be a consequence of knee extensor weakness or compromised knee extensor activity relative to body mass [17]. A reduced knee ROM was found also in swing phase. This result may be related to the relentless search for stability typical of obese individuals. As they aim at keeping both limbs in contact with the ground, this condition increases the amount of time spent in a closed lower-limb kinematic chain condition. In this situation, the degrees of freedom of the rigid lower body are reduced and the constraint, especially on the knee joint, increases. In addition, the excessive load on the limbs represents a source of further stress for the muscles involved in knee flexion movement during the pre-swing and swing phase. Then, another factor involved in the ROM reduction at the knee joint might originate from the excess of fat mass on the thigh and shank, which mechanically encumbers intersegmental rotation and counteracts the antigravity action exerted by the knee flexors [20].

On the frontal plane, higher values of the knee adduction are evident during the entire gait cycle. The literature is consistent in showing increased knee adduction in stance and swing phases [6,16], but not so in showing the effect of increased mass with respect to the effect of increased girths on gait. The increased step width is thought to be an obstructive mechanical issue related to the increased thigh girth [6,21], and it is possible that increased knee adduction is a consequential effect of the increased step width. This result is important from a rehabilitative point of view because an abnormal knee angle on the frontal plane may ultimately favor the development of osteoarthritis over time. As for the knee ab-adduction moment, the obese individuals walked with a significantly higher peak knee moment in absolute terms than controls. After normalizing the peak knee frontal plane moments by body weight, no significant difference was observed. Scanty, inconsistent evidence exists as for the knee moment on the frontal plane in literature [17]. We can speculate that the increased knee absolute ab-adduction moments found in this study could be related to the gait adaptations developed by the obese individuals over time. The latter may not be adequate to compensate for alterations in the frontal plane, leading to increased loads on the medial compartment joint. Increased amounts of adipose tissue between the thighs may represent a contributing factor to the larger knee abduction moments reported in the present study. A study investigating the gait patterns of obese adults found that they walk with significantly greater step widths than normal-weight adults. The authors proposed that the increased step widths of the obese group may have been the result of excessive amounts of adipose tissue between the thighs, in addition to providing a larger base of support during walking [22]. The repetitive stresses on the knee joint structures related to the higher frontal plane excursion and moments during stance may have the potential of inducing damages to knee joint structures, pain, limited motion and therefore, disability.

As for the ankle joint, kinematic data revealed a reduced plantarflexion position during terminal stance, which lead to a reduction in ankle ROM. This result may be directly related to a possible weakness of plantarflexor muscles in obese subjects [23] and probably associated with reduced physical activity typical of obese individuals [24], other than the previously quoted excessive amount of fat tissue. These abnormal ankle kinematics together with muscle weakness relate to the ankle moment and power. In terms of ankle moment, the obese individuals are characterized by lower peak of ankle plantarflexor moment and power during terminal stance at push-off [6,10]. These results may be due to relative plantarflexor muscle weakness, as these muscles are partial contributors to ankle joint moments in the sagittal plane, resulting in decreased push-off ability.

However, several differences among studies are present in terms of age, degree of obesity and methodological issues (i.e., the use of a treadmill mounted force plate to measure kinetic by Browning and Kram [15]).

This study has some limitations. Firstly, the tested sample was composed of young adults. Both males and females were recruited for this study in order to improve the generalizability of the results. Combining male and female subjects, however, introduces a source of potential variability, as obesity modifies the body geometry by adding mass to different regions and dissimilar fat distribution in males and females could produce gender-related effects. However, with our sample, it was not possible to consider them separately; thus, potential differences in movement characteristics between males and females who are obese will need further study.

Another limitation is due to the poor stratification of the participants across the three classes of obesity, since Class 1 was scarcely represented. Our data showed that differences between Obesity Class 2 and Class 3 were evident only in terms of kinetic parameters before normalization to body weight. This suggests that, despite the presence of obesity negatively impacting knee and ankle kinematics and kinetics during walking as compared to normal weight, only some kinetic parameters at the knee and ankle level worsen as BMI increases. It must be considered, however, that normalizing to body weight does not take into account body volumes, adipose distribution or body composition, which could have accounted for such differences. The absolute kinetic values differing in C2 and C3 describe a progressive increase in knee and ankle power linearly with BMI. The small sample size and the low number of individuals, particularly in Class 1, may have influenced the obtained results. Future studies with more participants for each obesity class are needed for a deeper understanding of the effects of different degrees of obesity on walking, as some effects may be independent of the degree of obesity, while others may be related.

Skin movement and marker placement errors are potential confounders of movement data, especially in subjects who are obese. Several methods were employed in the current study in an attempt to minimize these errors. The same investigator placed all markers on all participants to account for differences in marker placement due to investigator or day [18]. Hence, we are confident that the differences observed between groups were minimally due to skin movement or to marker placement.

## 5. Conclusions

In this study, differences in knee and ankle kinematics and kinetics during gait, between obese and normal-weight young adults, were identified. Our results might serve as a basis for developing targeted orthosis for individuals with obesity. Orthotics can be an effective complementary rehabilitative strategy for alleviating pain and improving function in obese subjects. Specific devices may help reduce pain at the knee and foot level caused by excessive weight. However, despite little doubt that orthotics able to withstand high forces on knee, feet and ankle due to excessive body weight would be beneficial and that pain relief would encourage engagement in more regular physical activity and favor weight loss, we have to consider that the same high forces acting on the joints and the orthosis might reduce their effectiveness, comfort or both. In fact, subjects with high degrees of obesity commonly encounter difficulties in identifying commercially available orthotics suited for them.

## Figures and Tables

**Table 1:** Anthropometric and clinical features of participants. Values are expressed as mean (SD).

Participants (M, F)	Age (years)	BMI (kg/m^2^)	Height (m)	Body Mass (kg)
CG (6, 10)	31.07 (5.45)	21.21 (1.99)	1.70 (0.06)	61.59 (8.47)
C1 (2, 0)	35.06 (6.98)	33.61 (1.88)	1.73 (0.12)	100.59 (6.42)
C2 (6, 11)	28.38 (6.63)	37.56 (1.05)	1.65 (0.09)	102.31 (11.95)
C3 (7, 6)	27.52 (8.13)	42.90 (2.11)	1.66 (0.11)	119.08 (18.31)

CG: Control Group; C1: Class 1: BMI of 30 to <35 kg/m^2^; C2: Class 2: BMI of 35 to <40 kg/m^2^; C3: Class 3: BMI of 40 kg/m^2^ or higher.

**Table 2:** Kinematic and kinetic variables, with mean (SD) in OG and CG. * *p* < 0.05.

Parameters	OG (32 Individuals)	CG (16 Individuals)
*Kinematic Variables (°)*		
KFE-IC	3.7 ± 4.0	4.4 ± 3.2
KFE-ROMSt	12.8 ± 4.4 *	14.9 ± 4.4
KFE-ROMSw	56.4 ± 6.9 *	61.2 ± 4.4
KRot-IC	−2.2 ± 8.0 *	−1.5 ± 9.5
MaxKRot	12.0 ± 7.7	14.5 ± 9.6
MaxKAASt	10 ± 4.3 *	−6.7 ± 8.8
MaxKAASw	28.0 ± 12.2 *	10.8 ± 7.5
ADP-IC	0.2 ± 3.2	−1.4 ± 2.4
MaxADP	12.8 ± 3.4	13.4 ± 2.8
minADP	−15.7 ± 5.8	−17.6 ± 5.5
ADP-ROM	28.5 ± 5.5 *	32.7 (4.4)
*Kinetic Variables*		
MaxKAAMom (N*m/kg)	0.6 ± 0.1	0.6 ± 0.1
MaxKAAMom abs (N*m)	60.9 ± 19.0 *	33.2 ± 7.5
MaxKFEMom (N*m/kg)	0.1 ± 0.1 *	0.4 ± 0.2
MaxKFEMom abs (N*m)	18.8 ± 11.9	21.1 ± 8.7
MaxADPMom (N*m/kg)	1.4 ± 0.2 *	1.5 ± 0.2
MaxADPMom abs (N*m)	160.0 ± 35.5 *	89.7 ± 15.5
minAP (W/kg)	−2.1 ± 0.3 *	−1.4 ± 1.2
minAP abs (W)	−110.5 ± 36.4 *	−48.2 ± 72.2
MaxAP (W/kg)	3.0 ± 0.6 *	3.4 ± 1.1
Max AP abs (W)	336.0 ± 74.1 *	202.7 ± 70.3

Legend: ROM: range of motion; IC: initial contact; Max: maximum; min: minimum; abs: absolute; KFE-IC: knee flex-extension at IC; KFE-ROMSt: knee flex-extension ROM in stance; KFE-ROMSw: knee flex-extension ROM in swing; KRot-IC: knee rotation at IC; MaxKRot: max knee rotation; MaxKAASt: max knee ab-adduction in stance; MaxKAASw: max knee ab-adduction in swing; ADP-IC: ankle dorsi-plantarflexion at IC; MaxADP: max ankle dorsi-plantarflexion; minADP: min ankle dorsi-plantarflexion; ADP-ROM: ankle dorsi-plantarflexion ROM; MaxADPMom: max ankle dorsi-plantarflexion moment; MaxKAAMom: max knee ab-abduction moment; MaxKFEMom: max knee flex-extension moment; minAP: min ankle power; MaxAP: max ankle power.

## Data Availability

Raw data are available in Zenodo. www.zenodo.org.
